# Relationship between Serum Levels of Metalloproteinase-8 and Tissue Inhibitor of Metalloproteinases-1 and Exercise Test Results in Postmenopausal Women

**DOI:** 10.1155/2016/7169531

**Published:** 2016-12-26

**Authors:** J. Mieczkowska, E. Rutkowska, J. Mosiewicz, B. Mosiewicz

**Affiliations:** ^1^Department of Internal Diseases, Medical University of Lublin, Lublin, Poland; ^2^Department of Tourism and Recreation, University of Life Sciences in Lublin, Lublin, Poland; ^3^Department of Internal Diseases, Students Medical Associations, Medical University of Lublin, Lublin, Poland

## Abstract

Physical activity as a part of the lifestyle is a significant factor influencing health condition. Exercises that require stamina are of particular importance. Oxygen metabolism, which is a significant part of all longer training processes, has an influence on cardiovascular and respiratory system functioning as well as all the processes taking part in maintenance of efficient homeostasis. Presentation of the correlation between exercise test results and MMP-8 (metalloproteinase-8) and TIMP-1 (tissue inhibitor of metalloproteinases-1) levels was attempted in this work. MMP-8 is a proteolytic enzyme taking part in progression of diseases related to process of ageing. 62 healthy women in postmenopausal period were qualified for the study (mean age: 54 ± 3.6). There was exercise test on the treadmill according to Bruce's protocol performed. MMP-8 and TIMP-1 serum levels were measured. There was statistically important correlation between increased level of MMP-8 and increased level of TIMP-1 with lower results of exercise test observed. The conducted study provides further biochemical arguments for prophylactic role of physical activity, which lowers the risk of noninfectious diseases, typical for middle adulthood, by influencing physical capacity.

## 1. Introduction

Systematic physical activity improves expression of genes which influence health condition [1]. Knowledge of these mechanisms enables considering moderate physical activity as one of the main means of chronic diseases prevention. An adequately high level of physical capacity in various age groups is one of health state indicators [2]. The assessment of body's morphological adaptation to physical exertion is a basis of reliable health-related training planning and physical rehabilitation. Research on connections between physical condition and level of chosen biochemical parameters (e.g., enzymes) may provide more arguments for thinking of physical activity as a factor influencing functioning of the body in various stages of life.

The postmenopausal period is a special time in women's life. Aside from frequent occurrence of typical ailments [3, 4], risk of cardiovascular events, which is significantly lower in premenopausal period in comparison with men, significantly increases [5–9]. Insufficient level of estrogens, which occurs in women in postmenopausal period, did not turn out to be influential enough in cardiovascular diseases pathogenesis to fulfill hopes related to hormone replacement therapy [10]. In women in this period of life cardiovascular diseases risk factors, such as hypertension [11–13], psychological disorders [14–17], overweight and obesity, dyslipidemia, and low physical activity [18–21], are commonly observed. The consequence of low physical activity is decreased physical capacity, which leads to premature involution processes and occurrence of risk factors, which frequently cause diseases of affluence. There is a connection between maximal oxygen consumption, VO_2_max, and chronic diseases [2]. Research on connections between chosen physiological, structural, and biochemical parameters and active lifestyle allows the understanding of this relation.

## 2. Aim of the Study

The aim of the study is to assess the connection between physical capacity measured with exercise test and indicator of early vascular changes, increased matrix metalloproteinase (MMP), and its inhibitor activity, in clinically healthy women in postmenopausal period. Matrix metalloproteinases are the main proteolytic enzymes responsible for apoptosis and angiogenesis. They are involved in pathogenesis of diseases related to the process of ageing, such as arthritis, atherosclerosis, and neoplasms [22]. Assessment of the connection between physical capacity and level of metalloproteinase activity could show protective influence of physical activity on lowered risk of these diseases.

## 3. Material and Methods

The examined group consisted of 62 Caucasian women in the postmenopausal period at the age of 54.6 ± 3.6. Patients with medical history or clinical or laboratory evidence of serious or unstable disorders, such as coronary artery disease, stroke, or other cerebrovascular events, peripheral vessels diseases diagnosed earlier, heart failure, cardiomyopathy, mitral valve leaflet prolapse syndrome confirmed with echocardiography, preexcitation syndrome present in electrocardiography (ECG) or in medical history, left bundle branch block, myocardial bridge in medical history, musculoskeletal diseases not allowing the performance of the exercise test, diabetes or other severe systemic or organs diseases, clinical signs of hyperandrogenism, and thyroid disease, were excluded from the study. Women treated with hormone replacement therapy were also excluded from the study.

Methods used in the study included history taking, physical examination, exercise test, and assessing activity of metalloproteinase-8 (MMP-8) and tissue inhibitor of metalloproteinases-1 (TIMP-1) in serum. Women were defined as postmenopausal according to following criteria: (1) duration of amenorrhea > 12 months and (2) blood concentration of follicle stimulating hormone (FSH) > 30 IU/mL [23, 24]. Examination was performed in the Department of Internal Diseases, Medical University of Lublin, Poland.

Medical history data and physical examination results were registered in a questionnaire. Waist circumference was measured; body mass index (BMI) and waist-hip ratio (WHR) were calculated. Waist circumference was taken as the minimum circumference between the umbilicus and the xiphoid process and measured to the nearest 0.5 cm. BMI was calculated as weight in kilograms divided by the square of height in meters.

ECG exercise stress tests were performed with the treadmill stress test (GE Medical Systems, Freiburg, Germany) according to Bruce's protocol [25]. Test was discontinued after the pulse limit was reached or in case of chest pain, vertigo, dyspnoea, changes in ECG which indicated ischemia, dysrhythmia, ventricular or supraventricular tachycardia, gradual pressure decrease, increase of systolic pressure (SBP) above 260 mm Hg, increase of diastolic pressure (DBP) above 115 mm Hg, and bradycardia. In the exercise test assessment initial pressure, maximum systolic and diastolic pressure measured in exertion, increase of systolic and diastolic pressure, maximum heart rate, pulse increase, metabolic equivalent of task (MET), and duration of exertion were taken into consideration. Test was discontinued according to common rules; observation also included recovery period.

Metalloproteinase-8 (MMP-8) and tissue inhibitor of metalloproteinases-1 (TIMP-1) levels in blood serum were assessed with immunosorbent tests using ELISA method with R&D Systems kits (Minneapolis, MN 55413, USA). Blood samples at rest, before exertion, were taken between 10 a.m. and 1 p.m. Blood level of metalloproteinase-8 (MMP-8) was assessed with kit whose catalogue number was DMP800; tissue inhibitor of metalloproteinase (TIMP-1) level was assessed with kit with catalogue number DTM100. Producer's instructions were followed. In every case calibration curve was made. Examination results were assessed with III universal microplatelets reader Bio-Tek ELX 800. Blood levels of MMP-8 and TIMP-1 were measured in ng/mL.

Data were processed using Statistica 10 (StafSoft) computer programme. Data are shown as means ± standard deviation (SD). Correlations between selected parameters were calculated using nonparametric test (*R*-Spearman's rank correlation coefficient).

The procedures of investigation were in accordance with the ethical standards of the responsible committee on human experimentation (Committee on Bioethics, Medical University of Lublin, KE-0254/185/2006) and with the Helsinki Declaration of 1975, as revised in 1983.

## 4. Results and Discussion

### 4.1. Results

The group of 62 women in the postmenopausal life period, confirmed by FSH level higher than 30 IU/mL, was examined. Mean menopause duration was 4.7 ± 4.5 years. The anthropometric data of studied group are presented in Table 1. None of examined women showed systematic physical activity. Their BMI values (Table 1) are indirect evidence of rather low physical activity [26]. In exercise test performed on treadmill mean exertion load was 9 MET and mean exertion duration was 7.9 min (Table 2). It allowed reaching average 83.2% of maximal pulse; average pulse increase from the initial pulse at rest was 68/min.

**Figure 1:**
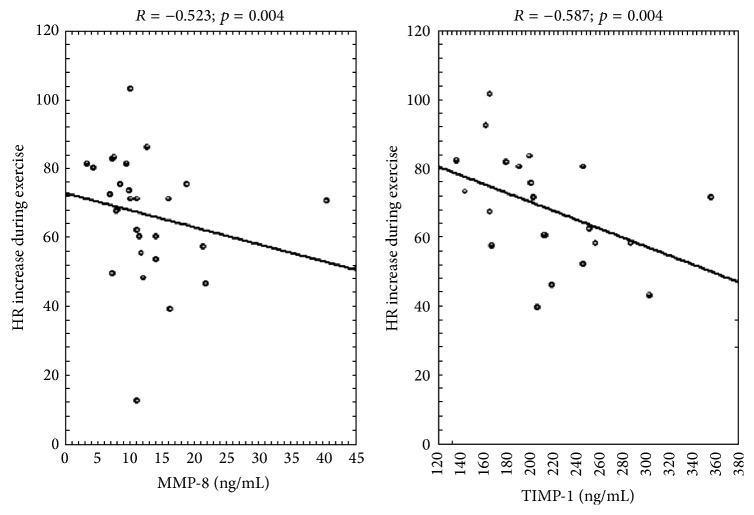
MMP-8 and TIMP-1 in relation to exertional heart rate increase. MMP-8: matrix metalloproteinase-8; TIMP-1: tissue inhibitor of metalloproteinases; HR: heart rate.

Mean metalloproteinase-8 level in blood serum in the examined group was 12.5 ± 7.5 ng/mL and mean tissue inhibitor of metalloproteinases-1 level was 221.7 ± 57.7 ng/mL. Concentration of MMP-8 in blood serum was statistically significantly negatively correlated with increase of heart rate during exertion test (*R* = −0.523; *p* = 0.004) (Figure 1). Positive correlation between MMP-8 level and diastolic pressure at rest was on the border of statistical significance (*R* = 0.343; *p* = 0.074). There were no statistical correlations between MMP-8 in serum and other exertion test parameters, MET, duration, and blood pressure (BP) in exertion and at rest (Table 3). TIMP-1 concentration in serum was significantly negatively correlated with heart rate increase (*R* = −0.587; *p* = 0.004) as well as maximal heart rate in exertion test (*R* = −0.432; *p* = 0.045). Other parameters of exertion test were not significantly correlated with TIMP-1 level in serum.

### 4.2. Discussion

Research on connection between active lifestyle and chosen physiological, structural, and biochemical parameters contributes to recognizing mechanisms of chronic diseases, such as cardiovascular diseases, which are the main cause of death and disability in group over 50 years old. Physical training improves the endothelium function [27] and is one of the most important cardioprotective means [28].

Clinical course of ischemic heart disease in women in postmenopausal period is specific. More often there are microcirculatory abnormalities, whose sign is positive electrocardiographic exercise test with no significant hemodynamic changes in the epicardial arteries. What is more, false positive and false negative results of the exercise test are more frequent, what limits usefulness of this examination in diagnostics of ischemic heart disease among women in postmenopausal period [29]. Exertion test may be a useful tool in planning health-related training; thus, it may be used in cardiovascular diseases primary prevention, particularly in groups with increased risk. One of these groups is women in postmenopausal period, independently from discussions about hormonal changes influence on the circulatory system. Physical capacity can be determined in the exertion tests using several measured parameters: heart rate at rest, its increase during exercise, blood pressure at rest and during exercise, and, finally, calculated metabolic equivalent of task.

One of the cardiovascular risk factors is low physical activity, which may modify exercise test results by influencing physical capacity of women after menopause. That is why the question regarding whether there is a correlation between exercise test results and early biochemical indicators of pathological process within the arteries remains to be answered. These indicators include metalloproteinases, enzymes which take part in extracellular matrix remodeling. They are secreted as proenzymes and activated by proteinases, plasmin, trypsin, chymotrypsin, kallikrein, cathepsin, and some MMPs. The main sources of all metalloproteinases are inflammatory cells [30, 31]. Metalloproteinases include cell membrane metalloproteinases which are related to cell membrane (MMP-14, MMP-15, MMP-16, and MMP-17), gelatinases (MMP-2 and MMP-9), stromelysins (MMP-3, MMP-7, MMP-10, MMP-11, and MMP-12), and collagenases (MMP-1, MMP-8, and MMP-13). Metalloproteinases take part in many physiological and pathological processes and they are responsible for remodeling and degrading the connective tissue [32]. MMP-8 is elevated in periodontal diseases in humans [33]. It is not specific and can be considered as general inflammation marker. However, there is a growing evidence suggesting a link between periodontal state and coronary heart disease [34]. Thus, a potential role of MMP-8 as a marker of increased risk of myocardial ischemia can be hypothesized.

Metalloproteinases activity may be moderated by tissue inhibitors, which include TIMP-1. Metalloproteinases inactivation is based on forming complexes which consist of active forms of MMP or some proenzymes and tissue inhibitor, complexes MMP-TIMP [35, 36]. TIMP-1 inhibits activity of majority of MMPs by forming irreversible complexes. Complexes MMP-TIMP take part in inflammatory processes, cardiomyocytes damage, forming aneurysms of the aorta, blood pressure regulation by influencing tension of arteries walls, vessels remodeling, coagulation, and angiogenesis processes [35–37].

In the atherosclerotic lesions MMP-8 expression was found. MMP-8 inactivation in experiments conducted on mice causes significant decrease of atherosclerotic lesions formation. Genetic knockout of MMP-8 in mice also results in lower angiotensin II concentration, lower blood pressure, and decrease of adhesive molecules number in atherosclerotic plaque, which lowers leucocytes adhesion to the endothelium. These data indicate an important role of MMP-8 in atherosclerosis process [37, 38]. That is why increased expression of MMP-8 along with decreased expression of its tissue inhibitor is disadvantageous and may be a sign of forming vessels changes of atherosclerotic nature, which is not necessarily clinically noticeable. In the studied group of women in postmenopausal period there was statistically significant correlation of MMP-8 increase in serum and small heart rate increase in exertion found. What is more, the disadvantageous changes in TIMP-1 were observed in the same group of patients. It is possible that higher heart rate at rest naturally resulting in lower difference between heart rates at rest and in exertion, which is characteristic for persons with lower physical activity and is one of atherosclerosis risk factors, causes tendency towards MMP-8 and its tissue inhibitor level increase in serum. What is more, disadvantageous changes in MMP-8 level seem to occur in patients with higher diastolic pressure at rest, though this correlation is on border of statistical significance, *p* = 0.074. Hence, MMP-8 level seems to be a potential candidate to a novel marker of cardiovascular risk among postmenopausal women.

To our best knowledge, there have not been any researches indicating correlations between exercise test results and metalloproteinases and their inhibitors blood levels in clinically healthy women in postmenopausal period yet. Confirmation of results of this study and their practical usefulness demand further research on bigger group of women.

## 5. Conclusions

1. Increased level of MMP-8 in serum is correlated with lower heart rate increase in exercise test in women in postmenopausal period.
2. Tissue inhibitor of metalloproteinases activity is higher among women in postmenopausal period who present lower maximal heart rate and lower heart rate increase in exertion.
3. These changes may constitute evidence of pathological process within the arteries in women with lower than common for this group physical capacity.

## Figures and Tables

**Table 1:** General characteristics of the examined group (*n* = 62) (mean ± standard deviation).

Age (years)	54.6 ± 3.6
Height (cm)	160.7 ± 4.8
Weight (kg)	71.5 ± 13.0
BMI (kg/m^2^)	27.7 ± 4.6
Waist circumference (cm)	89.4 ± 13.1
Waist-hip ratio	0.831 ± 0.060

**Table 2:** Exercise test results in examined group of women in postmenopausal period (*n* = 62) (mean ± standard deviation).

Exercise test duration (min)	7.9 ± 1.8
MET	9.0 ± 2.1
HR at rest (beats/min)	76.6 ± 11.8
SBP at rest (mm Hg)	127.4 ± 14.1
DBP at rest (mm Hg)	81.8 ± 10.5
HR reached during exercise (beats/min)	143.7 ± 17.4
HR increase during exercise (beats/min)	69.3 ± 23.0
HR max (beats/min)	172.8 ± 21.4
HR during exercise/HR max (%)	83.6 ± 12.3
SBP reached during exercise (mm Hg)	165.4 ± 17.1
DBP reached during exercise (mm Hg)	89.9 ± 9.0
SBP increase during exercise (mm Hg)	38.6 ± 18.0
DBP increase during exercise (mm Hg)	10.6 ± 8.9

SBP: systolic blood pressure; DBP: diastolic blood pressure; HR: heart rate; MET: metabolic equivalent of task.

**Table 3:** Exercise test parameters correlation with MMP-8 and TIMP-1 concentration in examined group of women in postmenopausal period (*n* = 62).

	MMP-8 (ng/mL)		TIMP-1 (ng/mL)	
	*R*	*p*	*R*	*p*
Exercise test duration (min)	−0.237	0.226	−0.138	0.551
MET	−0.103	0.601	−0.326	0.138
HR at rest (beats/min)	0.187	0.340	0.120	0.594
HR reached during exercise (beats/min)	−0.047	0.814	−0.432^*∗*^	0.045
HR increase during exercise (beats/min)	−0.523^*∗*^	0.004	−0.587^*∗*^	0.004
SBP at rest (mm Hg)	0.224	0.252	−0.074	0.744
DBP at rest (mm Hg)	0.343	0.074	−0.262	0.239
SBP reached during exercise (mm Hg)	−0.090	0.650	−0.076	0.738
DBP reached during exercise (mm Hg)	0.306	0.113	0.074	0.744
SBP increase during exercise (mm Hg)	−0.225	0.270	0.203	0.379
DBP increase during exercise (mm Hg)	0.074	0.718	0.153	0.508

SBP: systolic blood pressure; DBP: diastolic blood pressure; HR: heart rate; MET: metabolic equivalent of task.

^*∗*^Statistically significant.

## References

[1] Chakravarthy M. V., Joyner M. J., Booth F. W. (2002). An obligation for primary care physicians to prescribe physical activity to sedentary patients to reduce the risk of chronic health conditions. *Mayo Clinic Proceedings*.

[2] Bouchard C., Shephard R. J., Stephens T. (1994). *International Proceedings and Consensus Statement. Physical Activity, Fitness and Health*.

[3] (1996). Research on the menopause in the 1990s. Report of a WHO Scientific Group. *World Health Organization Technical Report Series*.

[4] Kenemans P. (1999). Menopause, HRT and menopausal symptoms. *Journal of epidemiology and biostatistics*.

[5] Dosi R., Bhatt N., Shah P., Patell R. (2014). Cardiovascular disease and menopause. *Journal of Clinical and Diagnostic Research*.

[6] Barrett-Connor E. (2013). Menopause, atherosclerosis, and coronary artery disease. *Current Opinion in Pharmacology*.

[7] Collins P., Rosano G., Casey C. (2007). Management of cardiovascular risk in the peri-menopausal woman—a consensus statement of European cardiologists and gynaecologists. *Kardiologia Polska*.

[8] Shaw L. J., Bugiardini R., Merz C. N. B. (2009). Women and ischemic heart disease: evolving knowledge. *Journal of the American College of Cardiology*.

[9] Abbasi S.-H., Kassaian S.-E. (2011). Women and coronary artery disease. Part I: basic considerations. *Journal of Tehran University Heart Center*.

[10] Hulley S., Grady D., Bush T. (1998). Randomized trial of estrogen plus progestin for secondary prevention of coronary heart disease in postmenopausal women: Heart and Estrogen/progestin Replacement Study (HERS) Research Group. *The Journal of the American Medical Association*.

[11] Tandon V. R., Mahajan A., Sharma S., Sharma A. (2010). Prevalence of cardiovascular risk factors in postmenopausal women: a rural study. *Journal of Mid-Life Health*.

[12] Yanes L. L., Reckelhoff J. F. (2011). Postmenopausal hypertension. *American Journal of Hypertension*.

[13] Routledge F. S., McFetridge-Durdle J. A., Dean C. R. (2009). Stress, menopausal status and nocturnal blood pressure dipping patterns among hypertensive women. *Canadian Journal of Cardiology*.

[14] Moilanen J. M., Mikkola T. S., Raitanen J. A. (2012). Effect of aerobic training on menopausal symptoms-a randomized controlled trial. *Menopause*.

[15] Barnes J. N., Hart E. C., Curry T. B. (2014). Aging enhances autonomic support of blood pressure in women. *Hypertension*.

[16] Kuh D., Hardy R., Rodgers B., Wadsworth M. E. J. (2002). Lifetime risk factors for women's psychological distress in midlife. *Social Science and Medicine*.

[17] Deeks A. A. (2003). Psychological aspects of menopause management. *Best Practice & Research Clinical Endocrinology & Metabolism*.

[18] Tandon V., Mahajan A., Mahajan S., Sharma S. (2014). Effect of life-style modification on postmenopausal overweight and obese Indian women: A Randomized Controlled 24 Weeks Preliminary Study. *Journal of Mid-life Health*.

[19] Kemmler W., von Stengel S., Bebenek M., Kalender W. A. (2013). Long-term exercise and risk of metabolic and cardiac diseases: the erlangen fitness and prevention study. *Evidence-Based Complementary and Alternative Medicine*.

[20] Kelley G. A., Kelley K. S., Tran Z. V. (2004). Aerobic exercise and lipids and lipoproteins in women: a meta-analysis of randomized controlled trials. *Journal of Women's Health*.

[21] Hernández-Ono A., Monter-Carreola G., Zamora-González J. (2002). Association of visceral fat with coronary risk factors in a population-based sample of postmenopausal women. *International Journal of Obesity and Related Metabolic Disorders*.

[22] Lipka D., Boratyński J. (2008). Metalloproteinases. Structure and function. *Postępy Higieny i Medycyny Doświadczalnej*.

[23] Randolph J. F., Zheng H., Sowers M. R. (2011). Change in follicle-stimulating hormone and estradiol across the menopausal transition: effect of age at the final menstrual period. *The Journal of Clinical Endocrinology & Metabolism*.

[24] Randolph J. F., Crawford S., Dennerstein L. (2006). The value of follicle-stimulating hormone concentration and clinical findings as markers of the late menopausal transition. *Journal of Clinical Endocrinology and Metabolism*.

[25] Fletcher G. F., Balady G. J., Amsterdam E. A. (2001). Exercise standards for testing and training: a statement for healthcare professionals from the American Heart Association. *Circulation*.

[26] Szeklicki R., Stemplewski R., Osiński W. (2006). Relations between habitual physical activity and BMI, WHR and body composition in elderly men. *Human Movement*.

[27] Hambrecht R., Wolf A., Gielen S. (2000). Effect of exercise on coronary endothelial function in patients with coronary artery disease. *New England Journal of Medicine*.

[28] Berlin J. A., Colditz G. A. (1990). A meta-analysis of physical activity in the prevention of coronary heart disease. *American Journal of Epidemiology*.

[29] Alexander K. P., Shaw L. J., Delong E. R., Mark D. B., Peterson E. D. (1998). Value of exercise treadmill testing in women. *Journal of the American College of Cardiology*.

[30] Sato H., Takino T., Okada Y. (1994). A matrix metalloproteinase expressed on the surface of invasive tumour cells. *Nature*.

[31] Kang Y.-J., Kim W.-J., Bae H.-U. (2005). Involvement of TL1A and DR3 in induction of pro-inflammatory cytokines and matrix metalloproteinase-9 in atherogenesis. *Cytokine*.

[32] Dudziak J., Sienkiewicz W. (2006). Metalloproteinases and their role in atherogenesis. *Czynniki Ryzyka*.

[33] Mc Crudden M. T., Irwin C. R., El Karim I., Linden G. J., Lundy F. T. (2016). Matrix metalloproteinase-8 activity in gingival crevicular fluid: development of a novel assay. *Journal of Periodontal Research*.

[34] Alfakry H., Malle E., Koyani C. N., Pussinen P. J., Sorsa T. (2016). Neutrophil proteolytic activation cascades: a possible mechanistic link between chronic periodontitis and coronary heart disease. *Innate Immunity*.

[35] De Clerck Y. A., Darville M. I., Eeckhout Y., Rousseau G. G. (1994). Characterization of the promoter of the gene encoding human tissue inhibitor of metalloproteinases-2 (TIMP-2). *Gene*.

[36] Ikonomidis J. S., Jones J. A., Barbour J. R. (2007). Expression of matrix metalloproteinases and endogenous inhibitors within ascending aortic aneurysms of patients with bicuspid or tricuspid aortic valves. *Journal of Thoracic and Cardiovascular Surgery*.

[37] Xiao Q., Zhang F., Lin L. (2013). Functional role of matrix metalloproteinase-8 in stem/progenitor cell migration and their recruitment into atherosclerotic lesions. *Circulation research*.

[38] Laxton R. C., Hu Y., Duchene J. (2009). A role of matrix metalloproteinase-8 in atherosclerosis. *Circulation Research*.

